# Does previous presentation of verbal fluency tasks affect verb fluency performance?

**DOI:** 10.1590/S1980-57642016DN10100006

**Published:** 2016

**Authors:** Bárbara Costa Beber, Márcia L.F. Chaves

**Affiliations:** 1 Dementia Clinic, Neurology Service, Hospital de Clínicas de Porto Alegre (HCPA), Porto Alegre RS, Brazil; 2 Department of Internal Medicine, Faculty of Medicine, Universidade Federal do Rio Grande do Sul (UFRGS), Porto Alegre RS, Brazil

**Keywords:** verbal fluency, verb fluency, aging, elderly, neuropsychological test, neuropsychology, fluência verbal, fluência de verbos, envelhecimento, idoso, teste neuropsicológico, neuropsicologia

## Abstract

**Background:**

Performance on the verb fluency (VF) task may be influenced by administration procedures and demographic factors of each population.

**Objective:**

The aim of this study was to verify whether the previous administration of semantic and phonemic verbal fluency tasks can influence performance on VF; and to analyze the correlation of VF performance with education, age and type of errors in Brazilian healthy elderly.

**Methods:**

Sixty-two participants were subdivided into experimental (semantic and the phonemic fluency tasks were administered before the VF) and control groups (VF only). The total score and the types of errors on the VF task were determined. Additional information was computed for the correlational analysis.

**Results:**

VF performance did not differ statistically between experimental and control groups, but correlated positively with education and negatively with intrusions.

**Conclusion:**

The lack of influence of other verbal fluency tasks on performance of the VF task in elderly individuals allows the use of this order of administration. A strong influence of educational level on VF task performance reinforces the need for further studies in different populations.

## RESUMO

**Introdução:**

O desempenho na tarefa de fluência de verbos (FV) pode ser influenciado pelo modo de administração e por fatores sócio-demográficos de cada população.

**Objetivo:**

Este estudo teve como objetivo verificar se a administração prévia das tarefas de fluência verbal (fluência semântica e fonêmica) poderia influenciar no desempenho da FV, assim como analisar a correlação da FV com escolaridade, idade e tipos de erros em Brasileiros idosos saudáveis.

**Métodos:**

essenta e dois participantes foram divididos em grupo experimental (fluências semântica e fonêmica foram aplicadas antes da FV) e em grupo controle (apenas a FV foi aplicada). O escore total e os tipos de erros da FV foram verificados. Informações adicionais foram consideradas para a análise de correlação.

**Resultados:**

Não houve diferença estatisticamente significativa entre os grupos experimental e controle quanto ao desempenho da FV. A FV se correlacionou positivamente com a escolaridade e negativamente com as intrusões.

**Conclusão:**

A falta de influência de outras tarefas de fluência verbal no desempenho da FV em indivíduos idosos permite o uso dessa ordem de administração. Uma forte influência da escolaridade sobre o desempenho da FV reforça a necessidade de estudos em diferentes populações.

## INTRODUCTION

Verb Fluency (VF) is a task of verbal fluency that requests the subject to say as many verbs (or "things that people do") as they can within one minute. The classic verbal fluency tasks (semantic and phonemic) request the production of nouns or object categories.^1^ Patients with different types of frontal brain pathology exhibit worst performance on the VF than on other verbal fluency tasks. This dissociation has been described in patients with nonfluent Primary Progressive Aphasia, behavioral Frontotemporal Dementia, HIV-1 infection, schizophrenia and Parkinson's disease.^2^^-^ 12

Evidence of this dissociation raises the possibility of using the VF task as a marker of frontosubcortical impairment.^13^ Given this clinical applicability, the task has been used in neuropsychological investigations. However, the administration of the VF task, akin to other neuropsychological tasks, might be influenced by administration procedures and/or demographic factors of individual populations. Previous studies using different tasks of verbal fluency, administered the VF after other verbal fluency tasks.^6^^-^ 8 ^,^ 11 ^,^ 14 The previous administration of other verbal fluency tasks might influence VF scores. The application of the California Verbal Learning Test-II (CVLT-II) prior to verbal fluency tasks produced higher verbal fluency scores.^15^ Nonetheless, the effect of verbal fluency tasks presented before the VF task has not yet been studied.

The influence of age and education on the VF task has been previously evaluated.^16^^-^ 18 Education showed higher impact on performance than age. However, these studies were carried out in developed countries in populations with higher educational level. Investigations of performance in low-educated populations from underdeveloped or developing areas of the world are still lacking.

The aim of this study was to verify whether the previous administration of semantic and phonemic verbal fluency tasks can influence performance on the VF task in Brazilian healthy elderly. The correlation of education, age and type of errors with the VF task was also analyzed.

## METHODS

**Translation and cultural adaptation of verb fluency instructions.** The instructions of the VF task are pivotal for proper application of the task. The instructions have to be easily understandable in terms of language as well as cultural features of the population in which the VF will be carried out. 

In the present investigation, two researchers produced a previous translation of the VF task instructions from the original,^19^ which included all sentences of the original instructions. The original instructions are as follows:

*"I'd like you to tell me as many different things as you can think of that people do. I don't want you to use the same word with different endings, like eat, eating, eaten. Also, just give me single words such as eat, or smell, rather than a sentence. Can you give me an example of something that people do?"* (Any verb response is acceptable) If the response is acceptable: *"That's the idea. Now in one minute, tell me as many different things as you can think of that people do"*.

A pilot study was carried out for the application of the translated VF instructions in an elderly group of mixed educational level. The aim of this pilot study was to define a Brazilian Portuguese version with easily understandable instructions for all educational levels (from illiterate to higher education). The investigators administered the direct translation of the instruction to a group of participants. Many participants had difficulty understanding the passages "I don't want you to use the same word with different endings" as well as "give me single words...". This difficulty probably occurred because most participants had a lower educational level, leading to greater difficulty in understanding the linguistic aspects of words. Based on these results, we devised a simplified version containing the essential instructions to execute the task. In an application of this adapted version, the participants showed satisfactory comprehension of the task.

The final Brazilian Portuguese instructions were as follows:

*"Irei marcar um minuto, e durante este tempo você terá que dizer o máximo de palavras que significam coisas que podemos fazer, como por exemplo, comer e andar. Você entendeu?"* Se a resposta for sim: *"Dê-me um exemplo, então."* Se o exemplo estiver correto: *"Agora, iremos começar. Diga-me outras palavras que representam coisas que podemos fazer, além de comer e andar".* Se o exemplo dado estiver incorreto, dê a explicação novamente com mais um exemplo. Para pessoas com maior escolaridade podem ser utilizados os termos "verbo" ou "ação" para explicar a tarefa. 

The English translation of the Brazilian Portuguese instructions is:

*"I will set one minute and during this time you will have to tell me as many words that mean things that people do as you can, for example eat and walk. Do you understand?"* If the answer is yes: *"Give me an example*". If the example is correct: *"We are going to start now. Tell me other words that mean things that people do, besides eat and walk."* If the given example is incorrect, give the explanation again with one more example. In the case of individuals with high educational level, the expressions "verb" or "action" can be used to explain the task. 

**Participants.** Sixty-two elderly subjects were recruited from social groups near the clinical research center of the *Hospital de Clínicas de Porto Alegre* (HCPA) or drawn from relatives or companions of patients of the dementia clinic at the same hospital. The inclusion criteria were: Brazilian Portuguese as the first language, at least 60 years of age, and no cognitive complaints. Individuals reporting medical history of any neurological and/or psychiatric diseases, uncorrected visual or hearing problems, and Mini-Mental State Exam (MMSE) scores below the cut-off for education^20^ were excluded.

**Procedures.** Participants were randomly subdivided into experimental (EG) and control (CG) groups. The EG comprised 32 participants who were administered the semantic^21^^,^ 22 and phonemic fluency (FAS)^23^ tasks before administration of the VF task. Thirty participants comprised the CG to whom the VF task was administered alone. Educational level, age, gender and handedness were matched between groups to ensure group homogeneity. Demographic data were similar between groups (Table 1).

**Table 1.** Demographic and clinical data of participants.

**Variable**	**All participants(n = 62)**	**CG(n = 30)**	**EG(n = 32)**	**p value(CG vs EG)**
Sex (% female)	87.1%	90%	84.4%	0.509**
Age	70.55 (± 5.52)	70.60 (± 5.01)	70.50 (± 6.04)	0.944*
Education (years)	7.94 (± 5.20)	7.30 (± 5.47)	8.53 (± 4.94)	0.355*
Handedness (% right)	85,48%	93,30%	93.80%	0.947**
MMSE	27.16 (± 2.04)	27.53 (± 2.03)	26.81 (± 2.02)	0.167*

CG: Control Group; EG: Experimental Group; *t-test; **Chi-Square test.

The final score on each verbal fluency task was the total number of correct items produced during one minute. Perseverations (repetition or inflection of a previously generated response, including the participant's self-generated word as the example or the example used in the instructions) and intrusions (words from other grammatical classes or neologisms) were scored separately to verify the errors produced by the participants. 

This study was approved by the HCPA Research Ethics Committee and all participants gave written informed consent.

**Data analyses.** All analyses were performed using the Statistical Package for Social Sciences (SPSS) version 18. The continuous variables were expressed as mean, standard deviation, range, and median while categorical variables were expressed as absolute and relative frequencies. The one-sample Shapiro-Wilk test was conducted to evaluate normality. The *t-*test was performed for the continuous variables with normal distribution, and Mann-Whitney or Kruskal-Wallis test for the continuous variables without normal distribution. The categorical variables were analyzed using the Chi-square association test. Education, age, and type of errors were evaluated with correlational analyses (Spearman's correlation). Subsequently, education was classified into subgroups (low, medium and high). A critical alpha of .05 was employed for the analyses. 

## RESULTS

No significant difference was observed in VF performance between the CG and EG (Mann-Whitney test; p = 0.133) (Table 2). Likewise, intrusions (p = 0.670) and perseverations (p = 0.969) did not differ between groups (Table 2). Since the previous application of the verbal fluency tasks did not affect the VF, we used data from the total sample for subsequent analyses. The distribution of VF total correct score, intrusions and perseverations are given in Table 3. 

**Table 2.** Distribution and comparison of verbal fluencies in Control and Experimental Groups

**Variable**	**CG (n = 30)**		**EG (n = 32)**		
	**Mean (SD)**	**Median**	**Mean (SD)**	**Median**	**p-value**
Verb Fluency - Total Correct	12.73 (5.85)	12.50	10.78 (5.69)	11.00	0.133*
Verb Fluency - Intrusions	0.14 (0.47)	0.00	0.06 (0.25)	0.00	0.670*
Verb Fluency - Perseverations	0.68 (0.89)	0.00	0.94 (1.56)	0.00	0.969*
Semantic Fluency - Total Correct	-	-	15.53 (4.20)	14.00	-
Phonemic Fluency -Total Correct	-	-	28.09 (10.62)	29.50	-

CG: Control Group; EG: Experimental Group; *Mann-Whitney test.

**Table 3.** Distribution of Verb Fluency results (total score and errors) in the total sample of Brazilian elderly people.

	**Mean (SD)**	**Range**	**Median**
Total score	11.73 (5.80)	4-29	11.00
Intrusions	0.09 (0.35)	0-2	0.00
Perseverations	0.83 (1.33)	0-7	0.00

The total score on the VF correlated positively with education (rho = 0.616; p = 0.001) and negatively with intrusions (rho = -0.318; p = 0.05) using Spearman correlation. Age (rho = -0.055; p > 0.05) and perseverations (rho = -0.017; p > 0.05) did not correlate with VF scores. 

Participants were subdivided into 3 groups of education, low (0-5 years), medium (6-11 years) and high (≥ 12 years), to compare VF scores among groups. The high education group showed significantly higher scores than the low education group (p < 0.05) (Table 4). 

**Table 4.** Distribution of Verb Fluency results (total score) in Brazilian elderly people according to education.

	**Low education(n = 28)**	**Medium education(n = 18)**	**High education(n = 16)**
Mean	8.75	11.89	16.75
SD	3.78	4.36	6.80
Range	4-18	6-23	6-29
Median	7.50^a^	11.00^ab^	15.50^b^

Kruskal-Wallis test. Same letters in medians indicate no significant difference; different letters in medians indicate significant difference on post hoc test (p < 0.05).

## DISCUSSION

We conducted this investigation to verify whether previous application of verbal fluency tasks can influence VF performance, and to verify the correlation of education, age, and type of errors with VF scores in a sample of Brazilian elderly. The analysis of f semantic and phonemic verbal fluency performance was not the aim of the study, therefore the reverse order (verbal fluency after VF) was not investigated. The VF scores were similar in both groups, demonstrating no effect of the application of verbal fluency tasks prior to the VF task.

Verbal fluency tasks demand a fast search of words in the lexical-semantic storage. In addition, other cognitive processes are also important, such as working memory and executive functions.^24^ The VF task requires the production of verbs. Verbs are more complex than nouns because they have more inflexions, more syntactic relationships with other words in the sentence, are acquired later in the process of language acquisition, have a less hierarchic semantic network, a greater number of high frequency words and are associated with longer reaction times in naming than nouns.^25^ All these features together mean the VF task places greater demands on executive functions and involves more activation in the semantic network than other verbal fluency tasks.

Verbs are a grammatical class of words referring to concepts of actions and have a syntactic relationship with nouns. In the event of administration of semantic and phonemic verbal fluency tasks prior to the VF task, the activation caused by nouns (from semantic and phonemic fluency tasks) in the semantic or lexical networks might propagate and activate syntactic networks or concepts of actions possibly associated with the nouns from the previous fluency tasks. The prior application of verbal fluency tasks may therefore facilitate retrieval of a higher number of verbs in the last task as an effect of practice or priming, consistent with the concept of the semantic network theory^26^^,^ 27. On the other hand, the presentation of the verbal fluency tasks before the VF task might hamper performance on the latter task as a consequence of fatigue. However, no effect of the prior administration of the verbal fluency task on the VF task was found in this investigation.

We observed significant correlation of education with VF total scores, as well as a significant difference between high and low education subgroups. The median observed was 7.50 in the low group, 11.00 in the medium, and 15.50 in the high education group. Participants with high educational level produced a greater number of verbs on this task. The effect of education on the VF task has been demonstrated in elderly populations. However, these populations typically had a higher educational level than that of the present sample.^5^^,^ 14 ^,^ 16 ^-^ 18 Individuals with higher educational levels may be better prepared for the task by recruiting the grammatical framework needed to produce action words.^14^ Our findings demonstrated the effect of education in this particular cognitive task. Education as an indicator of cognitive reserve has been investigated in different clinical conditions and in normal aging, showing a protective effect.^24^ Furthermore, individuals with higher educational levels have greater cognitive reserve and consequently more neural connections, making it easier to integrate information and access the lexicon.

We also observed a significant negative correlation of intrusions with VF total scores. The more verbs generated, the fewer intrusions registered, i.e., better, faster grammatical class processing is associated with fewer errors. We assume that intrusions may reflect difficulty in understanding the instructions of the task or difficulty accessing the grammatical class required. Higher intrusions may be a sign of disruption of VF processing due to the aging process. However, this relationship needs to be better investigated by studying VF errors in different clinical conditions.

On the other hand, perseverations can represent memory difficulty or attempts to fill time when new words do not come to mind. However, we found no correlation of perseveration with VF performance in this sample of healthy elderly. 

The present investigation showed the lack of influence of other verbal fluency tasks on the performance of the VF task in elderly individuals, which allows the use of this order of administration in clinical and research settings. A strong influence of educational level on VF task performance was also observed, reinforcing the need for further studies in different populations. The cultural and demographic profile of North American or European populations is very different from those observed in developing countries such as Brazil. To our knowledge, apart from some studies in Brazilian patients with clinical conditions,^24^^,^ 29 ^-^ 31 this is the first study focusing on the application of the VF task in a healthy elderly Brazilian population. Future studies should explore VF performance in younger population and other neurological disorders.
